# Investigating the influence of diet diversity on infection outcomes in a bumble bee (*Bombus impatiens*) and microsporidian (*Nosema bombi*) host-pathogen system

**DOI:** 10.3389/finsc.2023.1207058

**Published:** 2023-08-17

**Authors:** Abraham Martinez, Austin C. Calhoun, Ben M. Sadd

**Affiliations:** School of Biological Sciences, Illinois State University, Normal, IL, United States

**Keywords:** diet diversity, infection, *Nosema bombi*, pollinator health, pollen, parasite, *Bombus*

## Abstract

Diet can have an array of both direct and indirect effects on an organism’s health and fitness, which can influence the outcomes of host-pathogen interactions. Land use changes, which could impact diet quantity and quality, have imposed foraging stress on important natural and agricultural pollinators. Diet related stress could exacerbate existing negative impacts of pathogen infection. Accounting for most of its nutritional intake in terms of protein and many micronutrients, pollen can influence bee health through changes in immunity, infection, and various aspects of individual and colony fitness. We investigate how adult pollen consumption, pollen type, and pollen diversity influence bumble bee *Bombus impatiens* survival and infection outcomes for a microsporidian pathogen *Nosema (Vairimorpha) bombi*. Experimental pathogen exposures of larvae occurred in microcolonies and newly emerged adult workers were given one of three predominantly monofloral, polyfloral, or no pollen diets. Workers were assessed for size, pollen consumption, infection 8-days following adult-eclosion, survival, and the presence of extracellular microsporidian spores at death. Pollen diet treatment, specifically absence of pollen, and infection independently reduced survival, but we saw no effects of pollen, pollen type, or pollen diet diversity on infection outcomes. The latter suggests infection outcomes were likely already set, prior to differential diets. Although infection outcomes were not altered by pollen diet in our study, it highlights both pathogen infection and pollen availability as important for bumble bee health, and these factors may interact at different stages of bumble bee development, at the colony level, or under different dietary regimes.

## Introduction

An organism’s diet, through its nutrition or other elements, can have a profound influence on its ecology, including biotic interactions (1). This includes important effects of diet on the outcomes of pathogen infection, including pathogen traits of infectivity and transmission and host traits of health and fitness. In humans, the link between malnutrition and infectious disease is longstanding, with deficiencies in the diet compromising immunity and thus resistance to infection (2). Variation in host diet can influence immunity (3–5) and infection outcomes relating to the levels of infection and survival tolerance to infection (5–7). Non-nutritional compounds can also reduce infection (8). Diets can enhance the growth of commensal microbes that compete with pathogenic bacteria, indirectly reducing the opportunity for infection, or promoting immunity (9). Conversely, a highly diverse diet can catalyze the growth of symbiotic bacteria to levels that lead to costly upregulation of immunity (10). Diet composition can also affect strategic life history trait shifts, such as investment in reproduction upon parasite infection (11). As shown by these examples above, the evidence for diet-dependence of infection outcomes is widespread, but we still lack knowledge of how ecologically relevant variation in diet, including diversity, may affect many important host-pathogen interactions.

The health of pollinators globally is currently of great concern. Agricultural and other land use changes combined with other factors like nutrition, changing climate, pathogens and pesticides have been touted as being responsible for declines of several species of insect pollinators, including bees (12, 13). These factors deemed detrimental to pollinator health will have negative impacts on important agricultural and natural ecosystem pollination, increasing the need to elucidate the mechanisms by which they affect pollinator populations. In addition, the factors highlighted above have the potential to interact with one another, potentially to the further detriment of pollinator health (12, 14–16). This work focuses on the potential interaction between adult diet and infection outcomes in a bumble bee and microsporidian pathogen system.

Floral resources available to bees, which dictate their diet, have the potential to influence individual development and condition but also the health of social bee colonies (17, 18). Bee nectar and pollen consumption are responsible for being the main source of carbohydrates and protein, respectively, with pollen also providing lipids and micronutrients (19). Different flowers provide nectar that varies in carbohydrates and organic compounds important for pollinator attraction and floral defense (20), and pollen that varies in protein, lipid, sterol and essential amino acids (21–24). The available resources for bees may vary in quality and quantity, and land use changes, such as intense agriculture, can decrease floral diversity (25).

Available quantity, quality and diversity of pollen resources for important bee pollinators may induce foraging stress and have other direct and indirect effects on individual and overall colony health. Under such conditions bees may be faced with increased energetic costs of foraging (26) and nutritional deprivation. For example, *Bombus impatiens* colony-level performance strongly correlates with total amount of pollen collected and total macronutrient quantity (27). In addition, in intense agricultural environments depauperate in floral diversity, foragers of the bumble bee *B. terrestris* return with less diverse pollen loads compared with more diverse areas, and the diversity of collected pollen is positively correlated with a surrogate of colony growth and weight (28). Pollen availability and type has also been shown to influence individual development, size and survival, queen oviposition, and colony development (29–35). Even if pollen diversity is not important per se, but rather individual components of the pollen diet influence bee health (24), pollen diet diversity can contribute to buffering against poor quality pollen (34) and allow for diet optimization (23, 36).

There are likely many connections between nutrition and bee pathogen infection, including interactions that influence health (37). For example, choice of floral resources may affect pathogen exposure (38) and landscape simplification altering diet breadth appears to affect pathogen prevalence (39). Conversely, pathogen infection may influence foraging behavior, and hence diet. *Bombus impatiens* foraging efforts are impaired, specifically in discriminatory learning of rewarding and nonrewarding flowers, under the stress of infection by the trypanosome *Crithida bombi* (40). However, the focus here is on how diet may interact directly or indirectly, through bee host physiology and condition, with pathogen infections.

Pollen feeding alters *C. bombi* development in bumble bees (*Bombus terrestris*), with important effects on colony-level transmission (41). In addition, an increase in pollen consumption may indirectly benefit the host without necessarily influencing pathogen clearance. Honey bees (*Apis mellifera*) fed higher pollen quantity showed higher *Nosema cerenae* infection intensities but also had higher survival (42). Diverse poly-floral pollen also increased honey bee tolerance to *N. ceranae* infection above all but the most protein rich mono-floral diets (43). A similar benefit of poly-floral pollen was seen for honey bee larvae infected with an opportunistic fungal pathogen *Aspergillus fumigatus* (44). High diet diversity in honey bees increases glucose oxidase activity, a social immune measure important for an antimicrobial secretion in larval food and honey (45). Pollen diet quality can also affect bumble bee immunity (46), and immune gene expression in response to a trypanosome infection are reduced following pollen deprivation (47). Some pollen types, such as sunflower, have also been declared medicinal due to reductions in bumble bee trypanosome infection (48–50). These effects may not come from nutrition, but rather protective anti-microbial effects of phytochemicals in the diet (37). It is clear then, that both pollen diversity and pollen quality may be important for mitigating the negative effects of infection.

Many North American bumble bee species have declined in abundance and range over the last decades, with an apparent association between species declines and infection with the microsporidian pathogen *Nosema* (*Vairimorpha*) *bombi* (12, 51–53). Recently, there was a proposal to reclassify *Nosema* as *Vairimorpha* (54). However, while we acknowledge uncertainty in the classification, we maintain our use of the genus name *Nosema* given a lack of additional evidence that would be required for official reclassification (55). *Nosema bombi* has been shown to decrease bee fitness traits, including survival, sperm counts (56), and colony size (57). Adult workers deposit environmentally resistant spores in their feces (58), which contaminate nest material and floral resources. Larval stages are infected (59) and the intra-cellular pathogen stage hijacks ATP from host cells to develop and reproduce (60). Due to the drain by *N. bombi* on host energy reserves, infection outcomes may be expected to depend on nutritional resources, including those in the pollen diet. Although it has been shown that the isoflavanoid biochanin A, found in clover pollen, reduces *N. bombi* spores in adults but not larvae (61), no other studies have investigated such effects of bumble bee diet on infection with *N. bombi*.

There is limited understanding on how important pathogen and host parameters in chronic infections, including those of key pollinators, are influenced by diet diversity. In this study, we analyze the influence of adult pollen diet availability and diversity on outcomes of existing *N. bombi* infections in the bumble bee *B. impatiens*. We hypothesize that a pollen diet and the diversity of that diet will influence either resistance or tolerance to infection (62), based on the multiple stressor hypothesis of increased detrimental effects upon combined stressors (14). We use experimental pathogen inoculations during the larval stage combined with adult diets of no pollen, three predominantly mono-floral pollen diets (*Salix* sp., *Gleditsia triacanthos*, *Rubus* sp.) and one poly-floral pollen diet, combining these monofloral pollens, to assess the effects on diet consumption over time, pathogen spore loads, and host survival. We predict that pollen deprived bees and those fed mono-floral pollen diets will have lower resistance to infection (spore load) and that an interaction between pollen diet and *Nosema* infection on survival will be present because of further reduced tolerance to infection in pollen deprived and mono-floral pollen fed bees.

## Materials and methods

### Overview of experimental design to assess individual and combined pollen and pathogen effects

To assess individual and combined effects of diet treatment and *N. bombi* exposure and infection, developing larvae were either exposed or not exposed to a fixed number of spores during early development within their microcolonies. Subsequently, adults emerging in the microcolonies were isolated and provisioned with no pollen, one of three predominantly mono-floral pollen diets (confirmed molecularly as *Salix* sp., *Gleditsia triacanthos*, *Rubus* sp.), or a poly-floral diet consisting of an equal mix of the three mono-floral diets. Workers were assessed for effects of *Nosema* exposure or infection and pollen diet treatment on: (i) adult body size (n=200), (ii) pollen consumption (n=169), (iii) sporulating infection prevalence and spore count 8-days after adult emergence in a subset of bees killed at that time (n=76), (iv) infection prevalence and spore count at the time of death in a survival group and (v) their adult survival (n=166).

### Bumble bee source colonies

Microcolonies were established from ten commercial colonies (Koppert Biological Systems, Howell, Michigan, USA) and additional data for 8-day infection outcomes were collected from microcolonies sourced from a further three lab-reared colonies from field-caught queens. Queens of the lab-reared colonies were collected with the permission of the ParkLands Foundation (http://www.parklandsfoundation.org/) from the Mackinaw River Study Area (Lexington, IL., U.S.A.). All established colonies were confirmed free of common pathogen infections (including *N. bombi*) and maintained under standard laboratory conditions (14).

### *Nosema bombi* inoculum

Abdomens from three workers infected with a single *Nosema* isolate (lab identification: 017.01) were processed as in Calhoun et al. (14) and aliquots were stored at -80°C. When larvae were ready to receive the *Nosema* spore inoculum, aliquots were thawed on dry bath at 32°C. The aliquot was centrifuged at 3000 g for five minutes, and supernatant was removed. The pellet was then suspended in 1:1 v/v ultrapure water and 50% sugar water blended with five grains of fresh frozen pollen. Spore solutions were adjusted to 20,000 spores/μL for inoculation.

### Microcolony set-up and larval pathogen inoculation

Worker larvae in queenless microcolonies were designated to receive either *N. bombi* or a control solution containing only sugar water and pollen. Queenless microcolonies were constructed by randomly isolating five workers from a source colony into a plastic box with three medium petri dishes used to hold a brood clump of early instar (2^nd^) larvae and their surrounding wax that had been removed from the queen containing source colony, a standard poly-floral pollen pellet that was replaced three times per week, and a feeding tube providing sugar water *ad libitum*. All microcolonies were housed under red-light illumination at 26 ± 1.5°C.

Two-days after microcolony establishment, individual larva received a 2 μL inoculum of either 40,000 *N. bombi* spores suspended in a sugar water/pollen grain solution or a comparable solution without pathogen spores. Larval brood were temporarily separated from their respective microcolony, the wax of the larval casing was carefully peeled back utilizing soft forceps and a straight tip dissecting needle, exposing the larvae. Once uncovered, the 2 μL inoculum was delivered with a micropipette to the ventral side of each individual, allowing for the solution to adhere and be consumed by the larva. Each larval brood was left separated from the microcolony for five minutes to ensure full consumption of the treatment and placed back into their respective microcolony, where the wax was repaired by the nursing workers. This process was observed to ensure that larvae were not rejected. The original adult workers were marked with a spot of correction fluid on the top of the thorax and microcolonies were observed daily for adult emergence of the experimental individuals. Adults that emerged from each microcolony were removed within 24 hours and allocated uniformly to treatments within each replicate block. Removed individuals were isolated into plastic deli dishes (10 × 5 × 8 cm) with a 15 mL feeding tube containing sterile sugar water, paper substrate, and a 35mm petri dish with a pollen pellet for their respective diet treatment (see below). Deli dishes were replaced every 8 days for bees in the survival group. Body size from the radial cell length of the forewings for all individual (63) were measured using ImageJ software. The mean was taken from each pair of forewings for further analyses.

### Pollen diet treatments and consumption

Adults emerging from the microcolonies were provided with one of five pollen diet treatments. To produce three mono-floral diets, honey bee collected pollen loads were sorted based on color and the three most consistent and abundant were chosen (green, orange, yellow). A poly-floral blend was made by combining equal weights, to the nearest milligram, of the three mono-floral blends. Honey bees are known to predominantly collect one type of pollen per foraging trip (64). However, to confirm purity of mono-floral pollen diets and the diversity of the mono-floral versus poly-floral diets, four samples of each pollen type were suspended in ultrapure water and pollen grains counted in a FastRead 102 chamber under 400x magnification. All mono-floral diets had high levels of the focal pollen type, with the green 92% (s.d. 0.020%), orange 99% (s.d. 0.001%), and yellow 97% (s.d. 0.004%) purity. The Shannon Diversity Index, determined through microscopy for all pollen, the poly-floral treatment (1.20) was much higher than the green (0.27), orange (0.08) and yellow (0.17) mono-floral diets, indicating the desired difference between the treatments in diet diversity. The identity of the pollen was determined by DNA extraction from three replicates of individual pollen loads from each type using an IBI Scientific Mini Genomic Plant DNA Kit following the manufacturer’s instructions. PCR reactions were then set up using established primers and conditions for the *trnL*-*trnF* and ITS2 barcoding regions (65). All replicates for both regions were consistently identified as *Salix* sp. (Willow, orange), *Gleditsia triacanthos* (Honey locust, yellow), *Rubus* sp. (bramble, green) (**Supplementary Table 1**).

All pollen diets were made of the same ratio of sugar water to pollen and shaped into pellets using a 1 mL syringe, with the pollen cut every 100μl. Pellets were made fresh and replaced every 4 days. Consumption of diet treatments was measured by the difference in dry mass relative to the mean of 30 pre-weighed identically made reference pollen pellets of each diet. Dry weight was taken following a 2 day drying period in a drying oven at 60°C. All pellets were weighed on an XA Analytical Balance (Fisher Scientific) to the nearest milligram. A fifth pollen diet treatment consisted of no pollen, where workers were only fed sugar water *ad libitum*.

### Infection and survival responses

Infection was measured in a group of bees that were killed at eight-days post-eclosion to ensure a fixed timepoint for assessment of infection across individuals at a time when spores have been produced. These bees were stored at -20°C until processing. Whole bee abdomens were removed, homogenized in 1 mL ringer solution with an Omni TH_Q_ digital tissue homogenizer with a hard tissue tip. 10 μL of the homogenate was placed onto a hemocytometer and observed under a phase contrast microscope (Labomed Lx 500, Fremont, CA, USA) at 400x total magnification. Transmission ready spores were counted and converted to total spores per individual for further analysis. When spores are present, they are proportional to the overall infection intensity of intra-cellular stages (14).

Those bees assigned to the survival group were tracked daily for survival. Upon death, the date was recorded, and *Nosema* spores on death were quantified as above. Bees that did not die before the end of the experiment were included in analyses as right-censored observations.

### Statistical analyses

All analyses were performed in R version 3.6.3 “Holding the Windsock” for Mac (66). The *coxme* package (67) was used for Mixed Effect Cox Proportional Hazards Models and the *lme4* package for Generalized Linear Models (68). For each response variable, potential distributions were assessed for model fit and adherence to model assumptions. Initial models were simplified by sequentially eliminating non-significant terms based on likelihood ratio tests (LRTs) and nested models were selected using AIC (69). Statistics for terms not in final models were taken from the step before their removal. The package *emmeans* (70) was used to calculate estimated marginal means and their confidence intervals for levels of model terms and for *post hoc* contrasts.

For all models the original source colony and micro-colony, nested within original source colony, were included as random effects. A Linear Mixed Effects Model was fit with *Nosema* exposure treatment as a fixed effect to test the effect of larval exposure to the pathogen on adult body size. Additionally, a second model was fit only to the data from the *Nosema* exposed group to assess the influence of infection status, with the presence of spores when sampled taken as evidence of infection and the lack of spores as no infection. For a Linear Mixed Effects Model to analyze pollen consumption from 0 to 4 days and 4 to 8 days after adult eclosion, individual bee ID was included as an additional random effect to account for the repeated measures design. Further, the initial fitted model included body size, time period (0-4 and 4-8 days), *Nosema* exposure, pollen diet treatment, and all two-way interactions and the three-way interaction between time period, *Nosema* exposure, and pollen diet treatment. A second model was again fit with infection status within *Nosema* exposed bees replacing *Nosema* exposure treatment. Fixed time point infection prevalence at 8 days post-adult eclosion were analyzed based on binary infection prevalence (0/1). Infection prevalence was analyzed with a Generalized Linear Mixed Model fit with a binomial distribution and logit link function, including body size and diet in the initial model. The total number of spores was analyzed with a Linear Mixed Effects Model initially including body size and pollen diet treatment. The response variable of total spores was log transformed to meet model assumptions, and this approach was preferred as it produced a better fitting model than Generalized Linear Mixed Models with distributions including log link functions. These same analyses were repeated for spore presence and numbers at death in the survival group. A Mixed Effect Cox Proportional Hazards Model was used to evaluate the effect of body size, pollen treatment, *Nosema* exposure treatment, and the interaction between pollen and *Nosema* exposure treatments on individual survival. A similar model but only on individuals exposed to *Nosema*, replaced *Nosema* exposure treatment with infection status, as with body size and pollen consumption above.

## Results

### Body size

Comparing bees exposed as larvae to *Nosema* and unexposed bees, there was no significant difference in adult size (χ^2 =^ 0.316, d.f. = 1, p = 0.574). However, within *Nosema* exposed bees, those showing signs of infection (i.e., the presence of spores) were significantly smaller than those with no apparent infection (χ^2 =^ 4.01, d.f. = 1, p = 0.045) (**Supplementary Figure 1**).

### Pollen diet consumption

**Figure 1:**
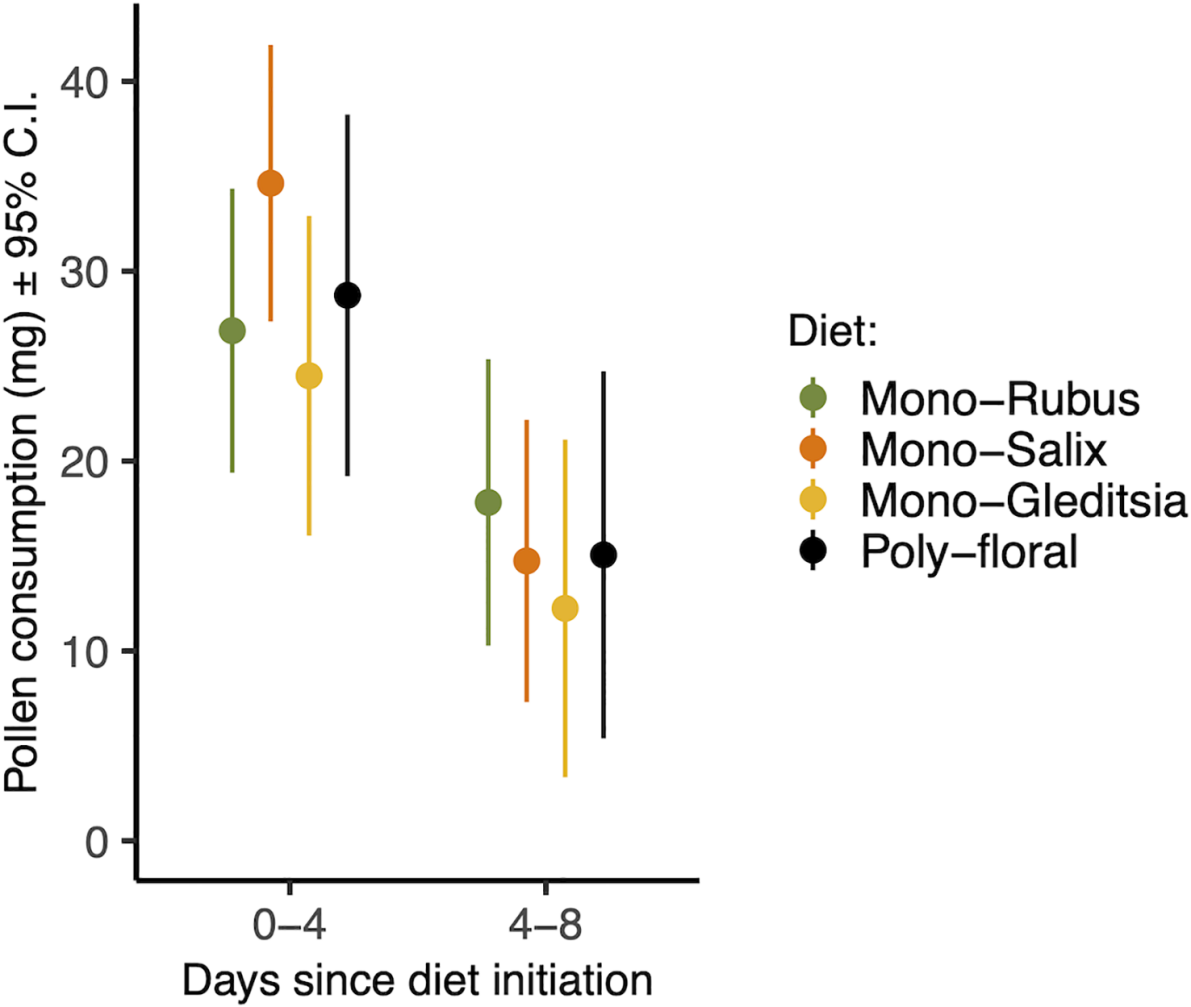
Total pollen consumed from days 0-4 and 4-8 for each pollen diet treatment. Points represent estimated marginal means and error bars represent 95% confidence intervals.

There was a significant effect of time period on pollen consumption (**Table 1**), with bees consuming more in their first four days than between day four and eight (**Figure 1**). Additionally, larger bees consumed more pollen (β = 0.346) (**Table 1**). There was, however, no effect of *Nosema* exposure, diet, or the interactions between them and time period on pollen consumption (p > 0.25) (**Table 1**). These outcomes did not change when comparing bees with evidence of infection and those without in the *Nosema* exposed group (**Table 1**).

**Table 1:** Fixed effect model terms for pollen diet consumption for (A) *Nosema bombi* exposed and unexposed bees, and (B) within *N. bombi* exposed bees, those with evidence of infection and those without.

Modelled responses	Terms	χ^2^	df	p
***(A) N. bombi exposed and unexposed***	**Body size**	**34.17**	**1**	**<0.0001**
	**Time period**	**28.98**	**1**	**<0.0001**
	Pathogen exposure	1.20	1	0.2732
	Pollen diet	2.11	3	0.5502
	Time period x Pathogen exposure	0.63	1	0.4284
	Time period x Pollen diet	2.79	3	0.4248
	Pathogen exposure x Pollen Diet	2.48	3	0.4788
	Time period x Pathogen exposure x Pollen diet	0.532	3	0.9119
***(B) Only N. bombi exposed***	**Body size**	**14.17**	**1**	**0.0002**
	**Time period**	**18.74**	**1**	**<0.0001**
	Infection status	0.34	1	0.5614
	Pollen diet	1.05	3	0.7895
	Time period x Infection status	0.17	1	0.6830
	Time period x Pollen diet	1.67	3	0.6432
	Infection status x Pollen Diet	1.43	3	0.6986
	Time period x Infection Status x Pollen diet	4.87	3	0.1816

Bolded terms were retained in the final model.

### Pathogen spores at 8 days after adult eclosion

**Figure 2:**
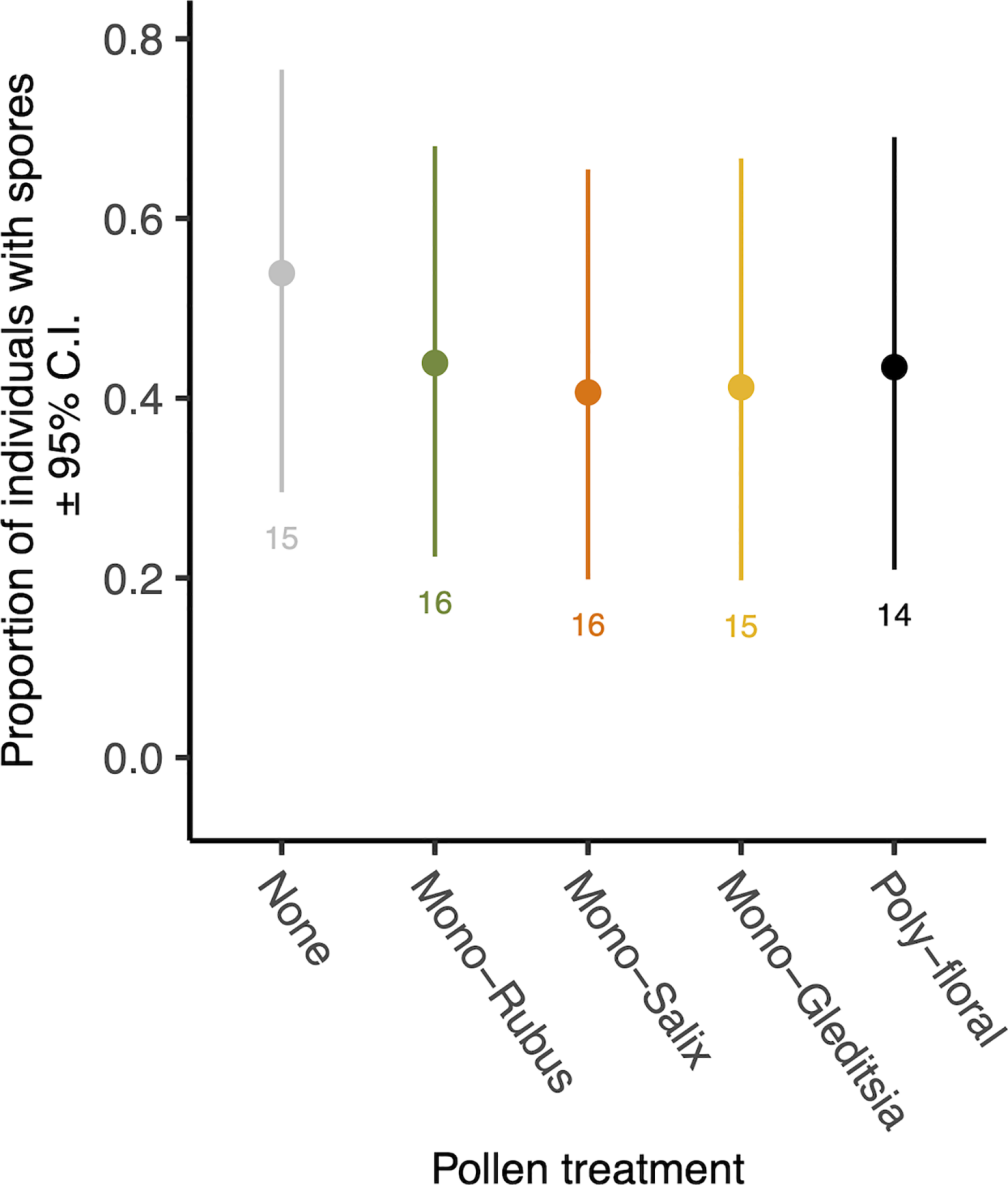
The proportion of 8-day post eclosion adult workers with *N. bombi* spores by pollen diet treatment. Points represent diet treatment estimated marginal means and error bars represent 95% confidence intervals. Numbers below bars represent sample sizes of each diet treatment.

At 8 days following adult eclosion, 45% of bees exposed to *Nosema* as larvae had spores present. There was no significant effect of the diet treatment on the presence of spores (χ^2 =^ 0.69, d.f. = 4, p = 0.953) (**Figure 2**), nor an effect of body size (χ^2 =^ 1.66, d.f. = 1, p = 0.198). Of those bees with spores present, there was no significant difference in spore numbers based on diet (χ^2 =^ 0.53, d.f. = 4, p = 0.970) (**Supplementary Figure 2**) or body size (χ^2 =^ 2.90, d.f. = 1, p = 0.088).

### Pathogen spores on death

**Figure 3:**
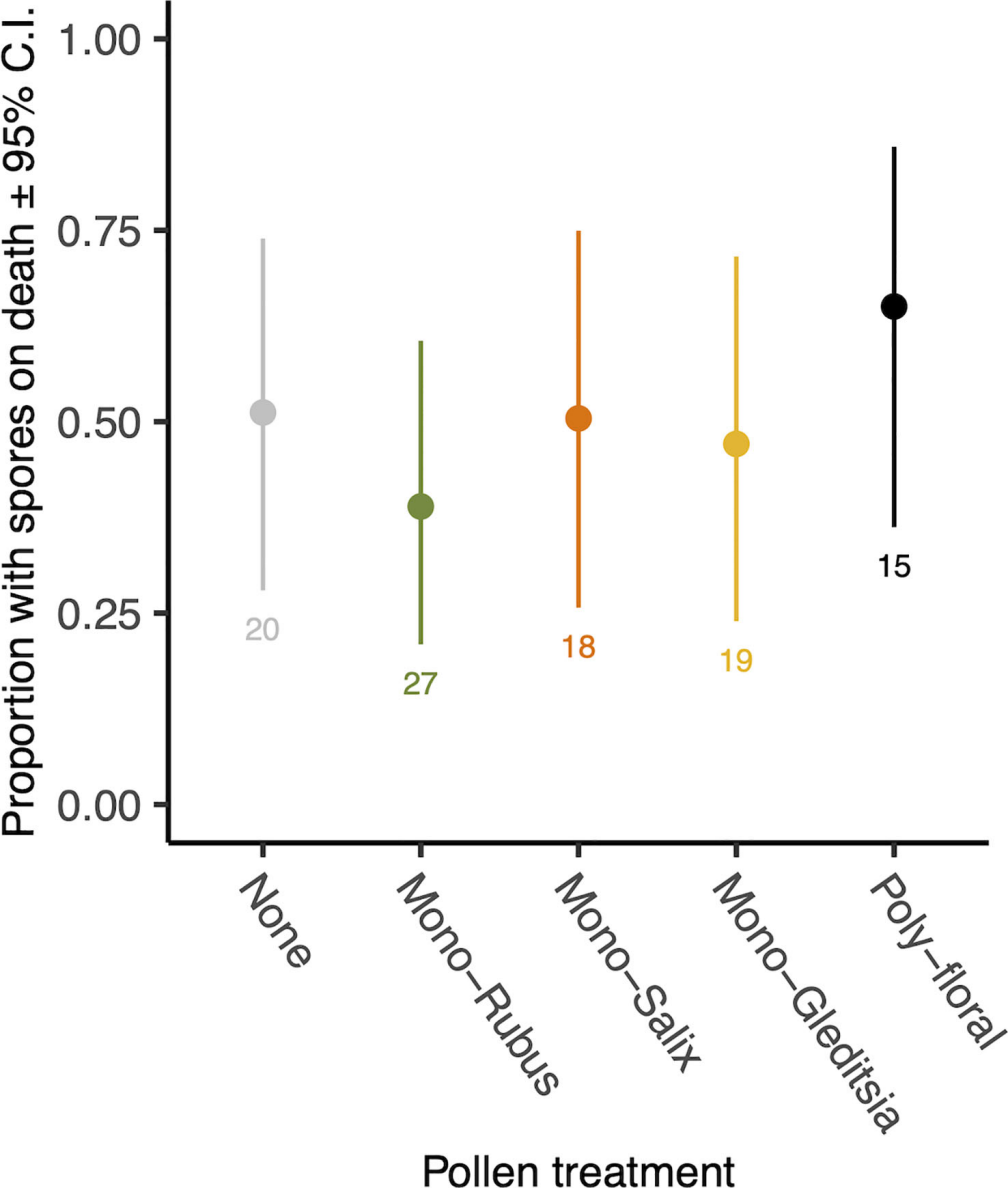
Proportion of workers with *N. bombi* spores at the time of death across pollen diet treatments. Points represent diet treatment estimated marginal means and error bars represent 95% confidence intervals. Numbers below bars represent sample sizes of each diet treatment.

At the time of their death, 47.5% of those bees exposed to *Nosema* had spores present. There was no effect of pollen diet treatment (χ^2 =^ 2.39, d.f. = 4, p = 0.664, **Figure 3**) or body size (χ^2 =^ 1.38, d.f. = 1, p = 0.240) on the proportion of bees with spores on death. Of those with spores present when they died, there was no significant difference in spore numbers based on diet (χ^2 =^ 2.06, d.f. = 4, p = 0.724) (**Supplementary Figure 3**). There was, however, a significant effect of body size (χ^2 =^ 5.35, d.f. = 1, p = 0.021), with larger bees having more spores when they died (**Supplementary Figure 4**).

### Survival

**Figure 4:**
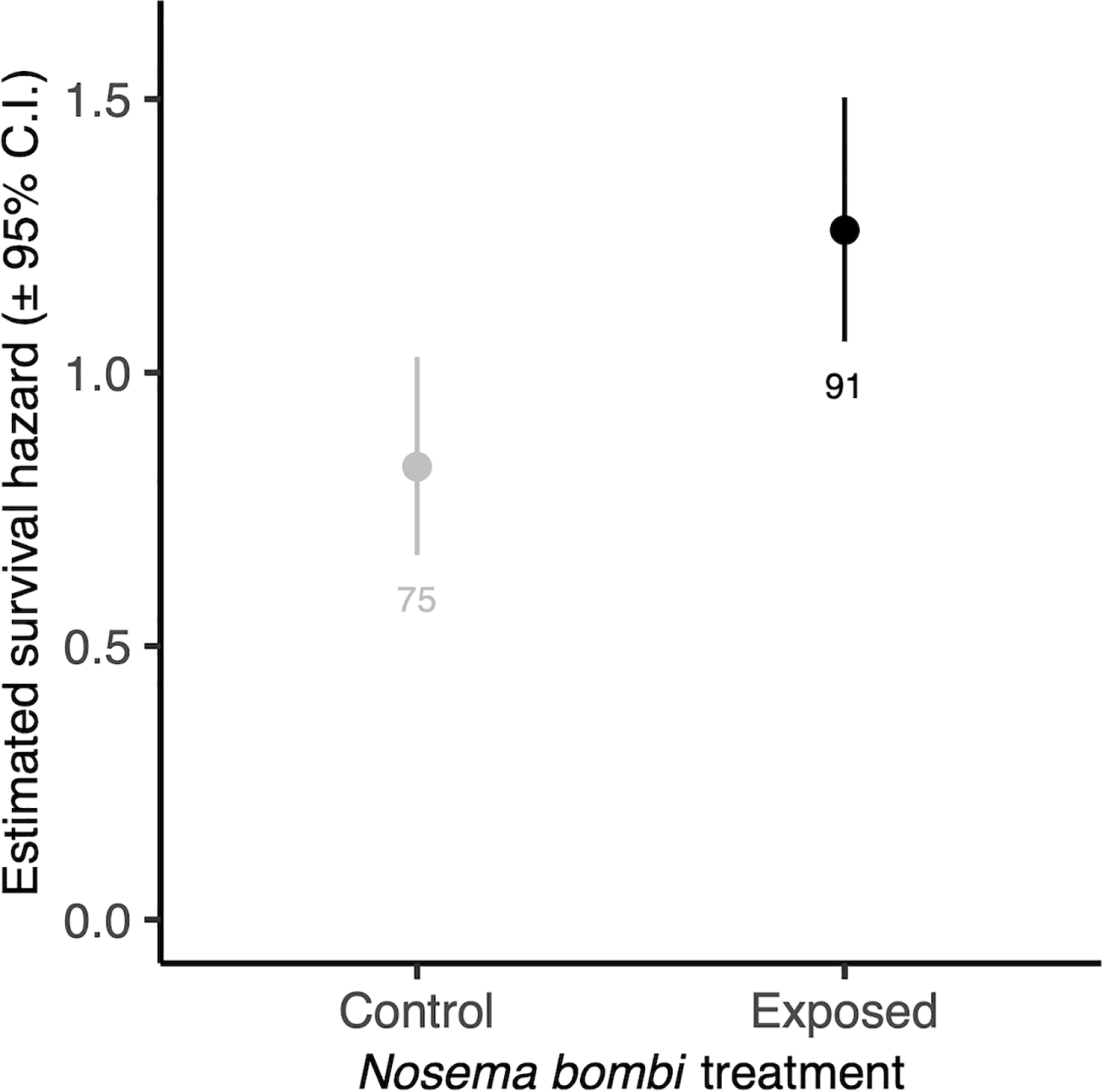
Estimated survival hazards of worker bees exposed to *N. bombi* and those not. Points represent model estimated survival hazard values and error bars represent 95% confidence interval. Numbers below bars represent sample sizes.

**Figure 5:**
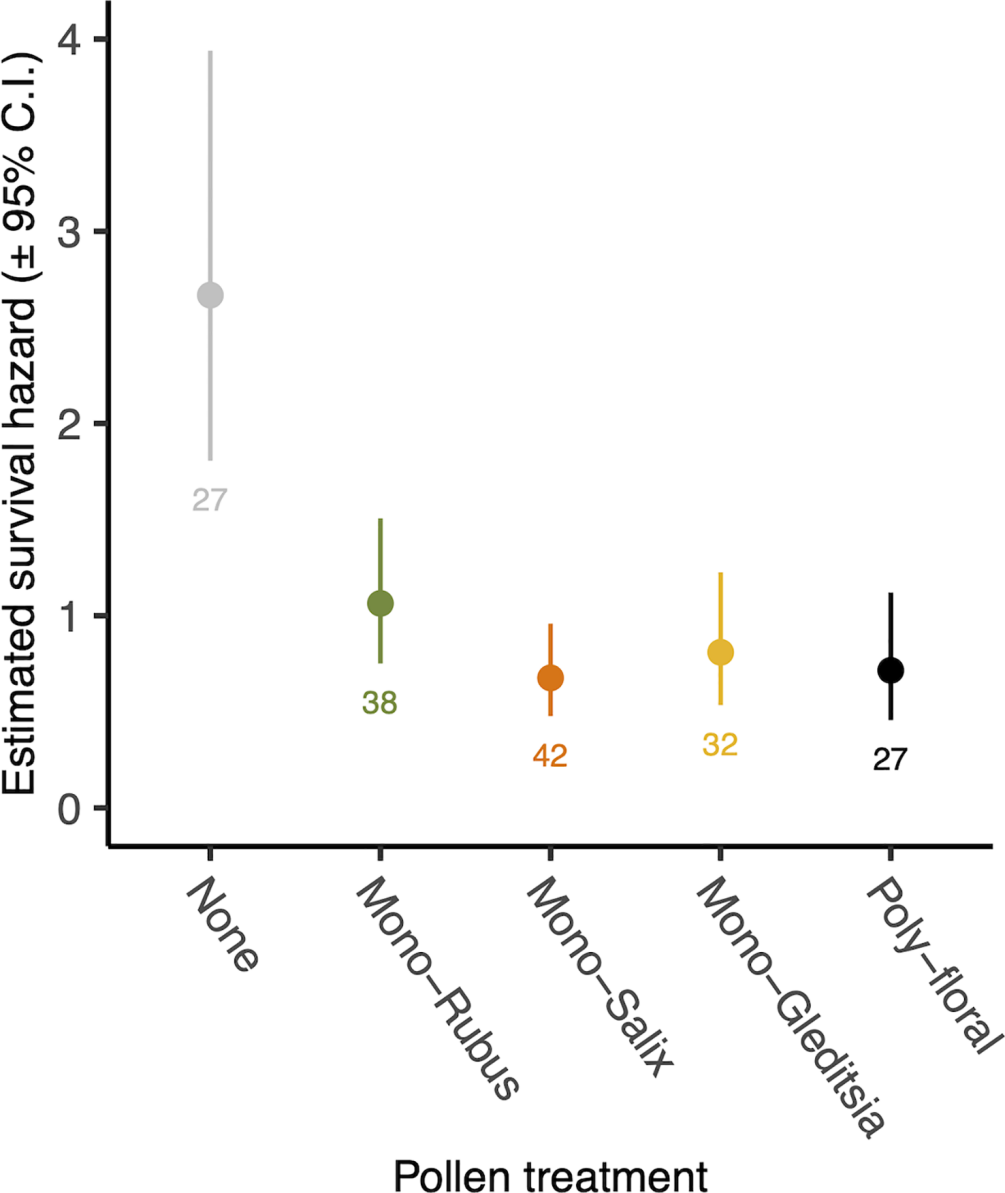
Estimated survival hazard of worker bees across pollen diet treatments. Points represent model estimated survival hazard values and error bars represent 95% confidence interval. Numbers below bars represent sample sizes.

There was no significant interaction between *Nosema* exposure and pollen diet on survival (χ^2 =^ 7.33, d.f. = 4, p = 0.119). However, pathogen exposed bees had a decreased likelihood of survival (χ^2 =^ 4.53, d.f. = 1, p = 0.033) (**Figure 4**). There was also a significant effect of pollen diet treatment on mortality (χ^2 =^ 27.40, d.f. = 4, p < 0.001, **Figure 5**), with *post hoc* tests indicating significantly increased probability of survival in individuals provided with pollen versus those not (Hazard ratio = 3.32, s.e. = 0.79, p < 0.001). Larger individuals also had a higher probability of survival (χ^2 =^ 5.62, d.f. = 1, p = 0.018). When only examining bees exposed to *Nosema*, and comparing bees with evidence of infection and those without, these outcomes were largely consistent for the interaction of pollen diet treatment and infection status (χ^2 =^ 1.75, d.f. = 4, p = 0.782), infection status (χ^2 =^ 3.97, d.f. = 4, p = 0.046), pollen diet treatment (χ^2 =^ 15.06, d.f. = 4, p = 0.005) and body size (χ^2 =^ 3.53, d.f. = 1, p = 0.060).

## Discussion

The pollen diet treatments employed in our study did not influence resistance to *N. bombi* infection, with prevalence of spores in eight-day adults, prevalence of spores upon death, and spore loads in dying bees not differing significantly across diet treatments. However, adult body size, something that can be affected by larval diet (31), affected some infection outcomes. *Nosema* infection negatively affected survival, but we did not find this to be affected by pollen diet treatment. However, pollen diet treatment itself significantly influenced survival, which was driven by reduced survival in pollen deprived adults. This supports other work suggesting the importance of pollen not just for larval development but also for adult bumble bee health (47, 71).

Contrary to our hypothesis, we did not find support that pollen diet, either pollen presence or diversity, influences *N. bombi* infection outcomes measured. This is in contrast to studies in honey bees infected with other *Nosema* spp. showing increased infection levels with increased pollen consumption (72, 73) and increased tolerance with greater pollen consumption (42) and with high diverse diets (43). Such effects may have been expected in bumble bees too, where the immune response to and infection by an adult bumble bee gut-infecting trypanosome, *C. bombi*, is affected in various ways by pollen availability and type (41, 47, 48). We suspect that in our study the exact infection outcomes of spore load and survival had been determined prior to the implementation of the different pollen diet treatments, given that infection establishment and development predominantly occur pre-adult. However, an interaction between pathogen infection and diet could be found under a different experimental regime. *Nosema bombi* infection establishes most successfully early in bumble bee development in larval instars (59). In our study, larval diet was consistent across all treatments, with pollen diet treatments only imposed during adulthood. It could be that by the time adulthood is reached, the trajectory of the infection is set. However, work showing that an isoflavonoid present in certain pollen types influences *N. bombi* spore load when consumed by adult but not larval bumble bees (61), suggests that this is not necessarily the case.

In addition to the temporal aspect between larval and adult diets, only three select pollen types were tested in our experiment, with pollen of *Salix* sp., *Gleditsia triacanthos*, and *Rubus* sp. In nature, a greater diversity or availability of different pollen may have different effects based on their distinct profiles of protein, lipid, amino acids, and secondary compounds. It is known that different pollen sources differ in important ways that may affect bumble bee individual and colony health (24, 33, 35), but a diverse poly-floral pollen diet is only expected to be beneficial if the mono-floral diets differ in parameters relevant for health. It has been previously shown that *Salix* and *Rubus* differ in protein:lipid ratios (19), but these differences were apparently not sufficient to drive different outcomes in terms of survival alone or infection outcomes documented. In nature, bumble bees may also regulate their intake of pollen in polyfloral landscapes to meet their nutritional demands (23, 27). If different infection outcomes were found under other varied pollen diets, it is feasible that bumble bees could balance their intake to minimize infection-related health losses, as has been seen in other insect systems (74, 75).

In addition to effects on infection resistance, the possibility that all individuals experienced similar diets during important stages of development may be an underlying reason that explains host survival in our study. Similar diets would result in similar anatomical development of Malpighian tubules, thorax muscles, fat body tissue and nerve tissues, all where *N. bombi* infects (76). The lack of any interaction between pollen diet treatment and infection is perhaps most surprising when contrasting the pollen provisioned and pollen deprived groups, given *N. bombi’s* metabolic need and energy depletion of host cells (60) and the reduced survival of pollen deprived bees. It is plausible that infected bees in the absence of pollen compensated the loss of energy through increased carbohydrate consumption through sugar water, which was provided *ad libitum* and was not measured in this study. Future studies should focus on varied pollen compositions and their influence during larval development on immunity and infection.

Although infection outcomes were not affected by diet, we observed negative effects on survival and adult body size in *N. bombi* infected bees. This study in *B. impatiens* adds to demonstrations from other bumble bee species of several detrimental impacts of individuals and colonies (56, 57, 77; but see 14). The documented effects on size and survival could come from the cost of infection directly due to tissue damage or energy utilization of the microsporidian (60, 76) or from a shift in resource allocation from development and other traits to upregulate costly immunity (78).

We found that larger bees had higher numbers of spores on death. An increased area in the gut has been proposed to be a potential cause for an increase in *N. bombi* infection (79), which could link body size, infection, and eventual spore numbers on death. However, a significant effect of size was not seen on infection at eight days post-adult eclosion. It is also possible that bees surviving longer could have more time for spores to accumulate. In honey bees, *N. ceranae* spore loads increased as bees aged (80). With survival positively associated with body size, the relationship between body size and spore number on death could be explained by a similar relationship between age and spore loads.

In conclusion, we did not find that infections were mitigated in adult workers given pollen or given poly-floral diets compared to mono-floral diets. However, we found evidence that both factors are important for determining bumble bee health, but in the experimental infection and diet regimes assessed here they act independently. Nevertheless, combinations of the stressors of pathogens and a dearth of pollen in bumble bee populations will be more detrimental than each alone, due to their additive effects. Moreover, at the colony or bee pollinator community level, it is plausible that pollen diets could interact with infection or dietary effects on pathogen infection could emerge. With anthropogenic stressors threatening floral resources, and therefore pollen availability, future work should further investigate such potential diet and infectious disease links.

## Data availability statement

The original contributions presented in the study are included in the article/**Supplementary Material**. Further inquiries can be directed to the corresponding author.

## Ethics statement

The manuscript presents research on animals that do not require ethical approval for their study.

## Author contributions

AM, AC, and BS conceived and designed the experiments. AM and AC performed the experiments. AM and BS analyzed the data. AM and BS wrote the manuscript; AC provided editorial advice. All authors contributed to the article and approved the submitted version

## References

[1] Raubenheimer D Simpson SJ Mayntz D. Nutrition, ecology and nutritional ecology: toward an integrated framework. Funct Ecol (2009) 23:4–16. doi: 10.1111/j.1365-2435.2009.01522.x

[2] Katona P Katona-Apte J. The interaction between nutrition and infection. Clin Infect Dis (2008) 46:1582–8. doi: 10.1086/587658 18419494

[3] Taylor CH Young S Fenn J Lamb AL Lowe AE Poulin B. Immune state is associated with natural dietary variation in wild mice *Mus musculus domesticus* . Funct Ecol (2019) 33:1425–35. doi: 10.1111/1365-2435.13354 PMC6767599 31588159

[4] Costantin EC Viol DL Del Puppo NP Elliot SL. Realism in immune ecology studies: artificial diet enhances a caterpillar’s immune defense but does not mask the effects of a plastic immune strategy. Front Insect Sci (2022) 1:754571. doi: 10.3389/finsc.2021.754571 PMC10926546 38468892

[5] Pirk CW Scheiner R. The effects of diet on health in insects. Front Insect Sci (2023) 3:1186027. doi: 10.3389/finsc.2023.1186027 PMC10926461 38469501

[6] Howick VM Lazzaro BP. Genotype and diet shape resistance and tolerance across distinct phases of bacterial infection. BMC Evolutionary Biol (2014) 14:56. doi: 10.1186/1471-2148-14-56 PMC3997931 24655914

[7] Frizzera D Ray AM Seffin E Zanni V Annoscia D Grozinger CM. The beneficial effect of pollen on *Varroa* infested bees depends on its influence on behavioral maturation genes. Front Insect Sci (2022) 2:864238. doi: 10.3389/finsc.2022.864238 PMC10926424 38468781

[8] Gowler CD Leon KE Hunter MD de Roode JC. Secondary defense chemicals in milkweed reduce parasite infection in monarch butterflies, *Danaus plexippus* . J Chem Ecol (2015) 41:520–3. doi: 10.1007/s10886-015-0586-6 25953502

[9] Jiang JF Song XM Wu JL Jiang YQ. Effects of alfalfa meal on the intestinal microbial diversity and immunity of growing ducks. J Anim Physiol Anim Nutr (2014) 98:1039–46. doi: 10.1111/jpn.12167 24460922

[10] Krams IA Kecko S Jõers P Trakimas G Elferts D Krams R. Microbiome symbionts and diet diversity incur costs on the immune system of insect larvae. J Exp Biol (2017) 220:4204–12. doi: 10.1242/jeb.169227 28939559

[11] Hudson AL Moatt JP Vale PF. Terminal investment strategies following infection are dependent on diet. J Evolutionary Biol (2019) 33:309–17. doi: 10.1111/jeb.13566 31705829

[12] Cameron SA Sadd BM. Global trends in bumble bee health. Annu Rev Entomol (2020) 65:209–32. doi: 10.1146/annurev-ento-011118-111847 31610137

[13] Winfree R Bartomeus I Cariveau DP. Native pollinators in anthropogenic habitats. Annu Rev Ecology Evolution Systematics (2011) 42:1–22. doi: 10.1146/annurev-ecolsys-102710-145042

[14] Calhoun AC Harrod AE Bassingthwaite TA Sadd BM. Testing the multiple stressor hypothesis: chlorothalonil exposure alters transmission potential of a bumblebee pathogen but not individual host health. Proc R Soc B: Biol Sci (2021) 288:20202922. doi: 10.1098/rspb.2020.2922 PMC8059969 33784861

[15] Vanbergen AJ The Insect Pollinators Initiative. Threats to an ecosystem service: pressures on pollinators. Front Ecol Environ (2013) 11:251–9. doi: 10.1890/120126

[16] Goulson D Nicholls E Botías C Rotheray EL. Bee declines driven by combined stress from parasites, pesticides, and lack of flowers. Science (2015) 347:1255957. doi: 10.1126/science.1255957 25721506

[17] Woodard SH. Bumble bee ecophysiology: integrating the changing environment and the organism. Curr Opin Insect Sci (2017) 22:101–8. doi: 10.1016/j.cois.2017.06.001 28805631

[18] Woodard SH Jha S. Wild bee nutritional ecology: predicting pollinator population dynamics, movement, and services from floral resources. Curr Opin Insect Sci (2017) 21:83–90. doi: 10.1016/j.cois.2017.05.011 28822494

[19] Vaudo AD Tooker JF Patch HM Biddinger DJ Coccia M Crone MK. Pollen protein: lipid macronutrient ratios may guide broad patterns of bee species floral preferences. Insects (2020) 11:132. doi: 10.3390/insects11020132 32085627 PMC7074338

[20] Heil M. Nectar: generation, regulation and ecological functions. Trends Plant Sci (2011) 16:191–200. doi: 10.1016/j.tplants.2011.01.003 21345715

[21] Tasei JN Aupinel P. Nutritive value of 15 single pollens and pollen mixes tested on larvae produced by bumblebee workers (*Bombus terrestris*, Hymenoptera: Apidae). Apidologie (2008) 39:397–409. doi: 10.1051/apido:2008017

[22] Weiner CN Hilpert A Werner M Linsenmair KE Blüthgen N. Pollen amino acids and flower specialisation in solitary bees. Apidologie (2010) 41:476–87. doi: 10.1051/apido/2009083

[23] Vaudo AD Patch HM Mortensen DA Tooker JF Grozinger CM. Macronutrient ratios in pollen shape bumble bee (*Bombus impatiens*) foraging strategies and floral preferences. Proc Natl Acad Sci U S A (2016) 113:E4035–42. doi: 10.1073/pnas.1606101113 PMC4948365 27357683

[24] Moerman R Vanderplanck M Fournier D Jacquemart AL Michez D. Pollen nutrients better explain bumblebee colony development than pollen diversity. Insect Conserv Diversity (2017) 10:171–9. doi: 10.1111/icad.12213

[25] Shuaib M Ali K Ahmed S Hussain F Ilyas M Hassan N. Impact of rapid urbanization on the floral diversity and agriculture land of district Dir, Pakistan. Acta Ecologica Sin (2018) 38:394–400. doi: 10.1016/j.chnaes.2018.04.002

[26] Heinrich B. Energetics of pollination. Annu Rev Ecol Systematics (1975) 6:139–70. doi: 10.1146/annurev.es.06.110175.001035

[27] Vaudo AD Farrell LM Patch HM Grozinger CM Tooker JF. Consistent pollen nutritional intake drives bumble bee (*Bombus impatiens*) colony growth and reproduction across different habitats. Ecology and Evolution (2018) 8:5765–76. doi: 10.1002/ece3.4115 PMC6010792 29938091

[28] Hass AL Brachmann L Batáry P Clough Y Behling H Tscharntke T. Maize-dominated landscapes reduce bumblebee colony growth through pollen diversity loss. J Appl Ecol (2019) 56:294–304. doi: 10.1111/1365-2664.13296

[29] Génissel A Aupinel P Bressac C Tasei JN Chevrier C. Influence of pollen origin on performance of *Bombus terrestris* micro-colonies. Entomologia Experimentalis Applicata (2002) 104:329–36. doi: 10.1046/j.1570-7458.2002.01019.x

[30] Leza M Watrous K Bratu J Woodard SH. Effects of neonicotinoid insecticide exposure and monofloral diet on nest-founding bumblebee queens. Proc R Soc B: Biol Sci (2018) 285:20180761. doi: 10.1098/rspb.2018.0761 PMC6015844 29899072

[31] Sutcliffe GH Plowright RC. The effects of food supply on adult size in the bumble bee *Bombus terricola* Kirby (Hymenoptera: Apidae). Can Entomologist (1988) 120:1051–8. doi: 10.4039/Ent1201051-12

[32] Yoon HJ Kim SE Lee KY Lee SB Park IG. Oviposition and colony development of the bumblebees, *Bombus ignitus and B. terrestris* depending on different pollen. Int J Ind Entomol (2005) 11:99–105.

[33] Moerman R Roger N De Jonghe R Michez D Vanderplanck M. Interspecific variation in bumblebee performance on pollen diet: new insights for mitigation strategies. PloS One (2016) 11:e0168462. doi: 10.1371/journal.pone.0168462 28005943 PMC5179047

[34] McAulay MK Forrest JRK. How do sunflower pollen mixtures affect survival of queenless microcolonies of bumblebees (*Bombus impatiens*)? Arthropod-Plant Interact (2019) 13:517–29. doi: 10.1007/s11829-018-9664-3

[35] Watrous KM Duennes MA Woodard SH. Pollen diet composition impacts early nesting success in queen bumble bees *Bombus impatiens* Cresson (Hymenoptera: Apidae). Environ Entomol (2019) 48:711–7. doi: 10.1093/ee/nvz043 31173096

[36] Vaudo AD Stabler D Patch HM Tooker JF Grozinger CM Wright GA. Bumble bees regulate their intake of essential protein and lipid pollen macronutrients. J Exp Biol (2016) 219:3962–70. doi: 10.1242/jeb.140772 27742891

[37] Koch H Brown MJ Stevenson PC. The role of disease in bee foraging ecology. Curr Opin Insect Sci (2017) 21:60–7. doi: 10.1016/j.cois.2017.05.008 28822490

[38] Piot N Meeus I Kleijn D Scheper J Linders T Smagghe G. Establishment of wildflower fields in poor quality landscapes enhances micro-parasite prevalence in wild bumble bees. Oecologia (2019) 189:149–58. doi: 10.1007/s00442-018-4296-y 30406396

[39] Figueroa LL Grab H Ng WH Myers CR Graystock P McFrederick QS. Landscape simplification shapes pathogen prevalence in plant-pollinator networks. Ecol Lett (2020) 23:1212–22. doi: 10.1111/ele.13521 PMC7340580 32347001

[40] Gegear RJ Otterstatter MC Thomson JD. Bumble-bee foragers infected by a gut parasite have an impaired ability to utilize floral information. Proc R Soc B: Biol Sci (2006) 273:1073–8. doi: 10.1098/rspb.2005.3423 PMC1560267 16600883

[41] Logan A Ruiz-González MX Brown MJF. The impact of host starvation on parasite development and population dynamics in an intestinal trypanosome parasite of bumble bees. Parasitology (2005) 130:637–42. doi: 10.1017/S0031182005007304 15977900

[42] Jack CJ Uppala SS Lucas HM Sagili RR. Effects of pollen dilution on infection of *Nosema ceranae* in honey bees. J Insect Physiol (2016) 87:12–9. doi: 10.1016/j.jinsphys.2016.01.004 26802559

[43] Di Pasquale G Salignon M Le Conte Y Belzunces LP Decourtye A Kretzschmar A. Influence of pollen nutrition on honey bee health: do pollen quality and diversity matter? PloS One (2013) 8:e72016. doi: 10.1371/journal.pone.0072016 23940803 PMC3733843

[44] Foley K Fazio G Jensen AB Hughes WO. Nutritional limitation and resistance to opportunistic *Aspergillus* parasites in honey bee larvae. J Invertebrate Pathol (2012) 111:68–73. doi: 10.1016/j.jip.2012.06.006 22750047

[45] Alaux C Ducloz F Crauser D Le Conte Y. Diet effects on honeybee immunocompetence. Biol Lett (2010) 6:562–5. doi: 10.1098/rsbl.2009.0986 PMC2936196 20089536

[46] Roger N Michez D Wattiez R Sheridan C Vanderplanck M. Diet effects on bumblebee health. J Insect Physiol (2017) 96:128–33. doi: 10.1016/j.jinsphys.2016.11.002 27836801

[47] Brunner FS Schmid-Hempel P Barribeau SM. Protein-poor diet reduces host-specific immune gene expression in *Bombus terrestris* . Proc R Soc B: Biol Sci (2014) 281:20140128. doi: 10.1098/rspb.2014.0128 PMC4046403 24850921

[48] Giacomini JJ Leslie J Tarpy DR Palmer-Young EC Irwin RE Adler LS. Medicinal value of sunflower pollen against bee pathogens. Sci Rep (2018) 8:1–10. doi: 10.1038/s41598-018-32681-y 30258066 PMC6158195

[49] Fowler AE Sadd BM Bassingthwaite T Irwin RE Adler LS. Consuming sunflower pollen reduced pathogen infection but did not alter measures of immunity in bumblebees. Philos Trans R Soc B (2022) 377:20210160. doi: 10.1098/rstb.2021.0160 PMC9058531 35491606

[50] Figueroa LL Fowler A Lopez S Amaral VE Koch H Stevenson PC. Sunflower spines and beyond: mechanisms and breadth of pollen that reduce gut pathogen infection in the common eastern bumble bee. Funct Ecol (2023) 37:1757–69. doi: 10.1111/1365-2435.14320

[51] Cameron SA Lozier JD Strange JP Koch JB Cordes N Solter LF. Patterns of widespread decline in North American bumble bees. Proc Natl Acad Sci U S A (2011) 108:662–7. doi: 10.1073/pnas.1014743108 PMC3021065 21199943

[52] Cameron SA Lim HC Lozier JD Duennes MA Thorp R. Test of the invasive pathogen hypothesis of bumble bee decline in North America. Proc Natl Acad Sci U S A (2016) 113:4386–91. doi: 10.1073/pnas.1525266113 PMC4843438 27044096

[53] Malfi RL Roulston TAH. Patterns of parasite infection in bumble bees (*Bombus* spp.) of Northern Virginia. Ecol Entomol (2014) 39:17–29. doi: 10.1111/een.12069

[54] Tokarev YS Huang WF Solter LF Malysh JM Becnel JJ Vossbrinck CR. A formal redefinition of the genera *Nosema* and *Vairimorpha* (Microsporidia: Nosematidae) and reassignment of species based on molecular phylogenetics. J Invertebrate Pathol (2020) 169:107279. doi: 10.1016/j.jip.2019.107279 31738888

[55] Aime MC Miller AN Aoki T Bensch K Cai L Crous PW. How to publish a new fungal species, or name, version 3.0. IMA fungus (2021) 12:1–15. doi: 10.1186/s43008-021-00063-1 33934723 PMC8091500

[56] Otti O Schmid-Hempel P. *Nosema bombi*: a pollinator parasite with detrimental fitness effects. J Invertebrate Pathol (2007) 96:118–24. doi: 10.1016/j.jip.2007.03.016 17482641

[57] Otti O Schmid-Hempel P. A field experiment on the effect of *Nosema bombi* in colonies of the bumblebee *Bombus terrestris* . Ecol Entomol (2008) 33:577–82. doi: 10.1111/j.1365-2311.2008.00998.x

[58] Rutrecht ST Brown MJ. The life-history impact and implications of multiple parasites for bumble bee queens. Int J Parasitol (2008) 38:799–808. doi: 10.1016/j.ijpara.2007.11.004 18164709

[59] Rutrecht ST Klee J Brown MJF. Horizontal transmission success of *Nosema bombi* to its adult bumble bee hosts: effects of dosage, spore source and host age. Parasitology (2007) 134:1719–26. doi: 10.1017/S0031182007003162 17610765

[60] Williams BA. Unique physiology of host–parasite interactions in microsporidia infections. Cell Microbiol (2009) 11:1551–60. doi: 10.1111/j.1462-5822.2009.01362.x 19673893

[61] Folly AJ Stevenson PC Brown MJ. Age-related pharmacodynamics in a bumblebee–microsporidian system mirror similar patterns in vertebrates. J Exp Biol (2020) 223:jeb217828. doi: 10.1242/jeb.217828 32107305

[62] Råberg L Sim D Read AF. Disentangling genetic variation for resistance and tolerance to infectious diseases in animals. Science (2007) 318:812–4. doi: 10.1126/science.1148526 17975068

[63] Müller CB Blackburn TM Schmid-Hempel P. Field evidence that host selection by conopid parasitoids is related to host body size. Insectes Sociaux (1996) 43:227–33. doi: 10.1007/BF01242924

[64] Castiglioni S Astolfi P Conti C Monaci E Stefano M Carloni P. Morphological, physicochemical and FTIR spectroscopic properties of bee pollen loads from different botanical origin. Molecules (2019) 24:3974. doi: 10.3390/molecules24213974 31684124 PMC6864723

[65] Kamo T Kusumoto Y Tokuoka Y Okubo S Hayakawa H Yoshiyama M. A DNA barcoding method for identifying and quantifying the composition of pollen species collected by European honeybees, *Apis mellifera* (Hymenoptera: Apidae). Appl Entomol Zoology (2018) 53:353–61. doi: 10.1007/s13355-018-0565-9 PMC6060998 30100617

[66] R Core Team. R: A language and environment for statistical computing. Vienna, Austria: R Foundation for Statistical Computing (2020).

[67] Therneau T. Mixed effects Cox models in R. Release 2.2-16 (2020). Available at: https://CRAN.R-project.org/package=coxme.

[68] Bates D Mächler M Bolker B Walker S. Fitting linear mixed-effects models using lme4. J Stat Software (2015) 67:1–48. doi: 10.18637/jss.v067.i01

[69] Burnham KP Anderson DR Huyvaert KP. AIC model selection and multimodel inference in behavioral ecology: some background, observations, and comparisons. Behav Ecol Sociobiol (2011) 65:23–35. doi: 10.1007/s00265-010-1029-6

[70] Lenth R Singmann H Love J Buerkner P Herve M. emmeans: estimated marginal means, aka least-squares means. Release 1.4.5 (2020). Available at: https://CRAN.R-project.org/package=emmeans.

[71] Smeets P Duchateau MJ. Longevity of *Bombus terrestris* workers (Hymenoptera: Apidae) in relation to pollen availability, in the absence of foraging. Apidologie (2003) 34:333–7. doi: 10.1051/apido:2003026

[72] Rinderer TE Dell Elliott K. Worker honey bee response to infection with *Nosema apis*: influence of diet. J Economic Entomol (1977) 70:431–3. doi: 10.1093/jee/70.4.431

[73] Fleming JC Schmehl DR Ellis JD. Characterizing the impact of commercial pollen substitute diets on the level of *Nosema* spp. in honey bees (*Apis mellifera* L.). PloS One (2015) 10:e0132014. doi: 10.1371/journal.pone.0132014 26226229 PMC4520664

[74] Povey S Cotter SC Simpson SJ Wilson K. Dynamics of macronutrient self-medication and illness-induced anorexia in virally infected insects. J Anim Ecol (2014) 83:245–55. doi: 10.1111/1365-2656.12127 PMC4217384 24033221

[75] de Roode JC Hunter MD. Self-medication in insects: when altered behaviors of infected insects are a defense instead of a parasite manipulation. Curr Opin Insect Sci (2019) 33:1–6. doi: 10.1016/j.cois.2018.12.001 31358187

[76] Fries I De Ruijter AAD Paxton RJ da Silva AJ Slemenda SB Pieniazek NJ. Molecular characterization of *Nosema bombi* (Microsporidia: Nosematidae) and a note on its sites of infection in *Bombus terrestris* (Hymenoptera: Apoidea). J Apicultural Res (2001) 40:91–6. doi: 10.1080/00218839.2001.11101056

[77] van Der Steen JJ. Infection and transmission of *Nosema bombi* in *Bombus terrestris* colonies and its effect on hibernation, mating and colony founding. Apidologie (2008) 39:273–82. doi: 10.1051/apido:2008006

[78] Sadd BM Schmid-Hempel P. Principles of ecological immunology. Evolutionary Appl (2009) 2:113–21. doi: 10.1111/j.1752-4571.2008.00057.x PMC3352412 25567851

[79] Larsson JR. Cytological variation and pathogenicity of the bumble bee parasite *Nosema bombi* (Microspora, Nosematidae). J Invertebrate Pathol (2007) 94:1–11. doi: 10.1016/j.jip.2006.07.006 17005191

[80] Li W Evans JD Li J Su S Hamilton M Chen Y. Spore load and immune response of honey bees naturally infected by *Nosema ceranae* . Parasitol Res (2017) 116:3265–74. doi: 10.1007/s00436-017-5630-8 29104999

