# Supplementary material for: Investigating the influence of diet diversity on infection outcomes in a bumble bee (Bombus impatiens) and microsporidian (Nosema bombi) host-pathogen system
Source: Front Insect Sci. 2023 Aug 17;3:1207058. doi: 10.3389/finsc.2023.1207058 (PMC10926413; doi:10.3389/finsc.2023.1207058)
Supplement: Supplementary file 1 [file DataSheet_1.docx]

Supplementary Material

INVESTIGATING THE INFLUENCE OF DIET DIVERSITY ON INFECTION OUTCOMES IN A BUMBLE BEE (*BOMBUS IMPATIENS*) AND MICROSPORIDIAN (*NOSEMA BOMBI*) HOST-PATHOGEN SYSTEM

Abraham Martinez^1,2^, Austin C. Calhoun^1^, Ben M. Sadd^1*^

^1^ School of Biological Sciences, Illinois State University, Normal, Illinois, 61790, USA

^2^ Current address: Department of Cell and Developmental Biology, University of Colorado, Anschutz Medical Campus, Aurora, Colorado, USA

*** Correspondence:**Ben M. Sadd

[bmsadd@ilstu.edu](mailto:bmsadd@ilstu.edu)

**Supplementary Table 1.** Sequences and proposed identities based on NCBI blastn hits of samples of individual pollen treatments based on trnL-trnF and ITS2 barcoding regions (Kamo et al. 2018).

See additional excel file

**Supplementary Figure 1.** Infection with *N. bombi* (based on spore presence) and adult body size. Points represent estimated marginal means and error bars represent 95% confidence intervals. Numbers below error bars represent sample sizes.

**Supplementary Figure 2.** The number of *N. bombi* spores per worker bee in those with spores at 8-days post-adult eclosion across pollen diet treatments. Points represent diet treatment estimated marginal means and error bars represent 95% confidence intervals. Numbers below bars represent sample sizes within each diet treatment.

**Supplementary Figure 3.** The number of *N. bombi* spores per worker bee in those with spores at the time of death across pollen diet treatments. Points represent diet treatment estimated marginal means and error bars represent 95% confidence intervals. Numbers below bars represent sample sizes within each diet treatment.

**Supplementary Figure 4.** The number of *N. bombi* spores per worker bee in those with spores at the time of death as a function of body size. Points represent individual values, with the red line and gray shading the model prediction and 95% confidence intervals.
